# Off‐Label Uses of Deucravacitinib for Inflammatory Skin Conditions: Cases and Literature Review

**DOI:** 10.1155/crdm/9941426

**Published:** 2026-07-07

**Authors:** Alexandra Nigro, Isabel C. Silva, Benjamin Gerstein, Jasper Dayanan, Saakshi Khattri

**Affiliations:** ^1^ Department of Dermatology, Icahn School of Medicine at Mount Sinai, New York, New York, USA, mountsinai.org; ^2^ Department of Dermatology, Macon & Joan Brock Virginia Health Sciences at Old Dominion University Eastern Virginia Medical School, Norfolk, Virginia, USA; ^3^ Department of Dermatology, Rutgers New Jersey Medical School, Newark, New Jersey, USA, rutgers.edu

**Keywords:** dermatomyositis, deucravacitinib, inflammatory, off-label, pityriasis rubra pilaris

## Abstract

Deucravacitinib is an oral, selective, allosteric tyrosine kinase 2 (TYK2) inhibitor approved for the treatment of moderate‐to‐severe plaque psoriasis and psoriatic arthritis. Deucravacitinib may offer a more selective approach compared to traditional Janus kinase (JAK) inhibitors. Emerging data support the use of deucravacitinib in inflammatory dermatoses beyond psoriatic disease. We present our clinical experience using deucravacitinib off‐label in patients with challenging inflammatory skin disease and contextualize our findings within the current literature. We report three female patients aged 31–63 years with refractory inflammatory skin conditions, including two with dermatomyositis and one with pityriasis rubra pilaris (PRP). All patients had persistent disease despite multiple systemic therapies, such as oral corticosteroids (OCS), acitretin, azathioprine, mycophenolate mofetil (MMF), JAK inhibitors, intravenous immunoglobulin (IVIG), and/or rituximab. Deucravacitinib 6 mg daily was initiated as adjunctive or alternative therapy. Both patients with dermatomyositis experienced meaningful cutaneous improvement; one also demonstrated improved proximal muscle strength and a sustained response over 2 years without adverse events. The other showed gradual resolution of facial erythema but developed a mild acneiform eruption. The patient with PRP achieved marked improvement by 6 months and near‐complete remission by 10 months, without reported adverse events. This case report supports the potential efficacy and adequate tolerability of deucravacitinib as an off‐label option for refractory inflammatory dermatoses, specifically dermatomyositis and PRP. Larger prospective studies are needed to better define its long‐term safety and therapeutic role beyond psoriasis.

## 1. Introduction

The Janus kinase (JAK)‐signal transducer has been implicated in many inflammatory and autoimmune skin conditions through dysregulated signaling and increased proinflammatory cytokine expression. JAK inhibitors (JAKi) have shown efficacy in the treatment of these conditions, both in clinical trials and real‐world off‐label use [1]. TYK2 pairs with JAK1 to form a heterodimer leading to the expression of cytokines including IL‐12, IL‐23, and IFN‐1 [2]. Deucravacitinib is an oral, selective, allosteric TYK2 inhibitor that is FDA‐approved for the treatment of moderate‐to‐severe plaque psoriasis [2, 3]. Deucravacitinib is highly selective for the tyrosine kinase 2 (TYK2) subunit, leading to targeted inhibition of these cytokines and potentially fewer off‐target effects compared with JAKi [2–4].

Off‐label use of deucravacitinib includes a wide breadth of pathology including lupus, alopecia areata, lichen planus, systemic sclerosis, interstitial pneumonia, IBD, and potential uses for neurodegenerative disease and malignancy [5]. Examples of successful use of deucravacitinib off‐label have been seen in several cases of inflammatory dermatoses; for example, cutaneous lupus has displayed improvement with deucravacitinib treatment [6].

Among inflammatory dermatoses that may benefit from deucravacitinib, dermatomyositis (DM) and pityriasis rubra pilaris (PRP) represent challenging clinical cases with limited treatment options [7–9]. DM is an autoimmune inflammatory condition characterized by both cutaneous and muscular findings, such as classic heliotrope rash, Gottron papules, shawl sign, V sign, and proximal muscle weakness [7]. PRP is a rare, chronic papulosquamous disorder characterized by “follicular hyperkeratotic papules that coalesce into scaly, reddish orange–colored plaques, which may progress to erythroderma with well‐demarcated islands of normal skin” [8, 9]. In addition to the conditions above, case literature has shown preliminary success of deucravacitinib for both PRP and DM [10–15]. This article reports three patients with refractory cutaneous inflammatory conditions, including PRP and DM, who responded favorably to off‐label use of deucravacitinib.

## 2. Case Presentation

### 2.1. Case 1

**FIGURE 1:**
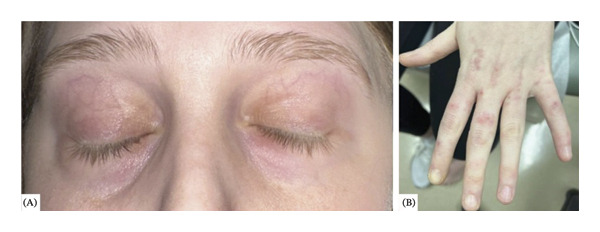
Baseline. Figure 1 depicts cutaneous manifestations of Case 1 at baseline, including facial skin involvement (A) and Gottron’s papules (B). Follow‐up images are unavailable for this case.

A 31‐year‐old Caucasian female with a 3‐year history of DM presented with +ANA speckled pattern 1:160, Gottron’s papules, heliotrope rash, and + biopsy and electromyography (Figure 1). The patient had trialed and had an inadequate response to MMF, azathioprine, and IVIG, and at the time of visit, she was on rituximab every 4 months along with upadacitinib. Physical examination showed hip flexor weakness and difficulty standing along with skin lesions. The patient reported frequent UTIs since starting upadacitinib; therefore, at the visit, upadacitinib was discontinued, and deucravacitinib 6 mg daily was added to rituximab every 4 months. At 3‐month follow‐up, there was improvement in muscle strength (3/5 power to 4/5 power) and skin clearance, and the patient reported improvement in breathing. The patient has been on deucravacitinib for 2 years now with no adverse events and sustained response.

### 2.2. Case 2

**FIGURE 2:**
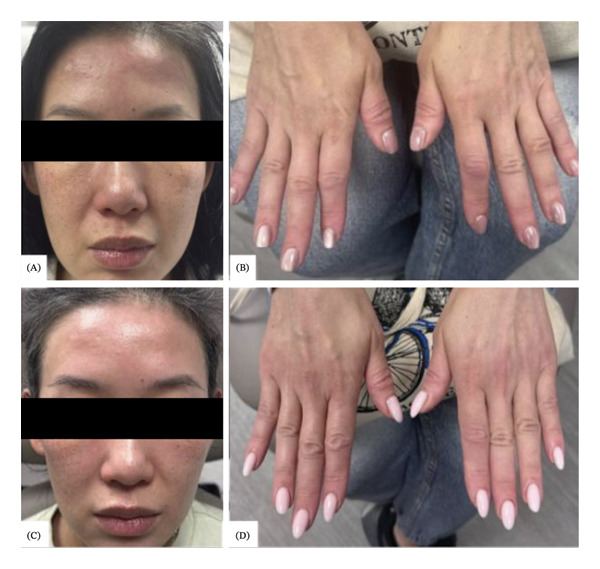
Baseline and 4‐month follow‐up. Figure 2 illustrates the cutaneous disease course in Case 2. Panels (A, B) show active dermatomyositis at baseline, prior to initiation of deucravacitinib. Panels (C, D) display facial skin and hands after 4 months of deucravacitinib therapy.

A 43‐year‐old Asian female with a history of facial rash for about a year had seen multiple dermatologists who treated her with topicals and oral prednisone but did not establish a clear diagnosis. On examination, she had diffuse erythema involving the face and dorsal hands, with flagellate erythema across the upper back (Figure 2). A biopsy and a MyoMarker panel were performed that showed perivascular infiltrate of mononuclear cells and ectatic vessels without epidermal changes and positive anti–MDA‐5 Ab. The patient was started on MMF 1.5 g BID and OCS. At 1‐month follow‐up, some improvement was noted on skin examination, but the patient remained concerned by persistent erythema, prompting the initiation of deucravacitinib 6 mg daily (Figure 2A). At one‐month follow‐up after starting deucravacitinib, the patient showed improvement of facial erythema. Subsequent follow‐up showed further improvement in facial erythema (Figure 2B). AEs included an acneiform eruption determined to be a side effect of the medication that led to discontinuation of deucravacitinib.

### 2.3. Case 3

**FIGURE 3:**
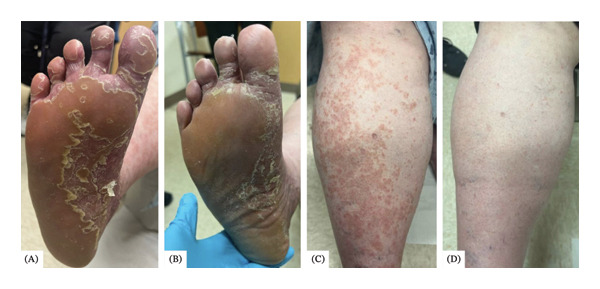
Baseline and 6‐month follow‐up. Figure 3 demonstrates treatment response in Case 3. Panel (A) shows active skin disease on the right plantar foot at baseline; Panel (B) shows the same region after 6 months of deucravacitinib therapy. Panels (C, D) show the left posterior calf at patient baseline and after 6 months of treatment, respectively.

A 63‐year‐old Caucasian female was referred from an outside provider with PRP on shins, feet, upper thigh, groin, and hands for a duration of 1 year. On physical examination, palmoplantar hyperkeratosis with orangish plaques on bilateral legs, feet, knees, groin, and upper inner thighs with islands of sparing was observed (Figure 3A, C). A diagnosis of PRP was made clinically based on physical examination findings. The patient had tried and failed topical steroids, OCS, and acitretin. The patient was initiated on once daily 6‐mg deucravacitinib as an off‐label therapeutic intervention. At 6‐month follow‐up visit, palmar and plantar plaques showed significant improvement (Figure 3B, D). At 10‐month follow‐up visit, the patient’s skin was almost clear with no AEs. At last follow‐up, the patient is in remission and had clear skin on examination.

## 3. Discussion

This case series presents three cases of off‐label use of deucravacitinib in recalcitrant inflammatory dermatoses, including PRP and DM. The use of deucravacitinib in DM has been supported by case literature with studies reporting efficacy in combination with IVIG, hydroxychloroquine, or MMF and even as monotherapy [10–12]. TYK2 polymorphisms have been linked to susceptibility for rheumatic disease, including DM, implicating TYK2 as a novel therapeutic target for DM [13]. Additionally, TYK2/JAK1 heterodimers drive downstream activation of IFN‐1, a key overexpressed cytokine in DM, providing further mechanistic support [2, 13].

For PRP, recent literature has supported the use of deucravacitinib, with near resolution of skin disease at 6 months in one case and 9 weeks in another [14, 15]. This aligns with aberrant immune activation involving the IL‐23/Th17 axis in PRP, pathways shared with psoriasis for which deucravacitinib is currently approved to treat [9]. A clinical trial is ongoing to assess this treatment for use in PRP [NCT06444399].

Though conventional systemic therapies exist for both conditions, many patients experience inadequate response, relapse, or intolerable adverse effect [7–9]. Compared with broader JAKi, deucravacitinib may offer a promising alternative given its greater selectivity within the JAK‐STAT signaling cascade [4]. This distinction is clinically meaningful, as it allows modulation of key inflammatory pathways while minimizing off‐target immunosuppression. Clinical trials in psoriasis have confirmed this favorable safety profile, with low rates of serious infections, laboratory abnormalities, thromboembolic events, or cardiovascular complications [3]. One mild AE was reported leading to drug discontinuation in one case, though deucravacitinib demonstrated clinical efficacy prior to cessation. These results support TYK2 inhibition as a targeted option that offers JAK‐like benefits with a potentially more favorable safety profile.

Limitations include small sample size, retrospective design, and limited follow‐up. Larger prospective and randomized studies are needed to clarify long‐term safety and efficacy.

## Funding

None of the funding acknowledged in the COI was utilized in preparing this manuscript or completing this research.

## Consent

The authors obtained written consent from patients for their photographs and medical information to be published in print and online and with the understanding that this information may be publicly available. Patient consent forms were not provided to the journal but are retained by the authors.

## Conflicts of Interest

Dr. Saakshi Khattri is an employee of Mount Sinai and receives research funds from Leo Pharma, AbbVie, Bristol Myers Squibb, Pfizer, Celgene, and Acelyrin. Dr. Saakshi Khattri is also a consultant for Leo, AbbVie, Eli Lilly, Janssen, Regeneron, Sanofi, and UCB.

The other authors declare no conflicts of interest.

## Data Availability

The data that support the findings of this study are available from the corresponding author upon reasonable request.
